# Quantification of prostate tumour diameter and volume from MR images using 3D ellipsoid model and its impact on PI-RADS v2.1 assessment

**DOI:** 10.1038/s41598-022-26065-6

**Published:** 2022-12-13

**Authors:** Dharmesh Singh, Chandan J. Das, Virendra Kumar, Anup Singh, Amit Mehndiratta

**Affiliations:** 1 Centre for Biomedical Engineering, Indian Institute of Technology Delhi, New Delhi, India; 2 Department of Radiodiagnosis, All India Institute of Medical Sciences, New Delhi, India; 3 Department of NMR, All India Institute of Medical Sciences, New Delhi, India; 4 Department of Biomedical Engineering, All India Institute of Medical Sciences, New Delhi, India; 5 Centre for Biomedical Engineering, IIT Delhi Hauz-Khas, Room No-298, Block III, New Delhi, 110016 India

**Keywords:** Cancer, Engineering

## Abstract

Maximum diameter and volume of the tumour provide important clinical information and are decision-making parameters for patients suspected with prostate cancer (PCa). The objectives of this study were to develop an automated method for 3D tumour measurement and compare it with the radiologist’s manual assessment, as well as to investigate the impact of 3D tumour measurement on Prostate Imaging-Reporting and Data System version-2.1 (PI-RADS v2.1) scoring of prostate cancer. Tumour maximum diameter and volume were calculated using automated ellipsoid-fit method. For all PI-RADS scores, mean ± standard deviation range of tumour maximum diameter and volume measured using ellipsoid-fit method were 1.36 ± 0.28 to 1.97 ± 0.67 cm and 0.49 ± 0.31 to 1.05 ± 0.78 cc and manual assessment were in range of 0.73 ± 0.12 to 1.14 ± 0.25 cm and 0.36 ± 0.21 to 0.93 ± 0.39 cc, respectively. Ellipsoid-fit method showed significantly (*p* < 0.05) higher values for maximum diameter and volume than manual assessment. 3D measurement of tumour using ellipsoid-fit method was found to have higher maximum diameter and volume values (in 40–61% patients) compared to conventional assessment by radiologist, which may have an impact on PI-RADS v2.1 scoring system.

## Introduction

Accurate measurement of tumour size in patients with prostate cancer (PCa) is becoming important to guide treatment planning^1^. Tumour maximum diameter (TMD) and tumour volume (TV) are well established prognostic factors to assess the risk of a clinically significant PCa (csPCa)^2–4^. Advances in MRI have provided a non-invasive method of evaluating TV, although this may be underestimated with measurement from MRI as was reported previously^5–7^. The overall underestimation of TV with MRI may be understood from the study of Langer et al.^6^, where histologic findings identified sparse areas of PCa containing normal tissue intermixed with malignant epithelium, which may not be distinguished on MR images.

Prostate Imaging-Reporting and Data System version 2 (PI-RADS v2) provides assessment scores for PCa on a scale of 1 to 5, in which score-5 is most likely to represent csPCa^8^. The revised PI-RADS v2.1 relies mainly upon subjective analysis of MRI findings with quantitative features, including: tumour-size, TV and mean apparent-diffusion coefficient (ADC)^9^. PI-RADS v2.1 incorporates a tumour-size based decision-criterion (cut-off = 1.5 cm) to differentiate between assessment of score-4 and score-5^9^. PI-RADS v2.1 recommends upon minimal requirements for the measurement of TV, whereas insists to report the maximum tumour diameter of a lesion on an axial images^9^. TMD represents the actual tumour dimension, which can be in any plane, where axial two-dimensional (2D) might or might not be the correct representation. Thus three-dimensional (3D) assessment of tumour is highly desirable for practical purposes^10^. Based on the applications of MRI, csPCa is defined as Gleason score > 7 and TV > 0.5 cc^9^. Martorana et al. found that as PI-RADS score increases the probability of detecting a csPCa proportionally increases with increase in TV^11^. Bratan et al. identified the accuracy of volume assessment was influenced by the size of lesions, Gleason score, and PI-RADS score^12^.

A recent review by Eldred-Evans et al. has reported an increasing number of studies evaluating volumetric assessment of tumours against radical prostatectomy as the reference standard^5^. Diameter-based tumour-size measurement has been the clinical standard method to assess maximum diameter and volume^13^. This method is easy and fast to perform in a busy clinical routine but may be less accurate because PCa may spread through the tissue along any axes^13^. Many methods have been used to determine TV, but computer-assisted image analysis based volume measurement is considered as the most accurate^14^. Planimetric calculation includes the contouring of the lesion on each axial-slice which puts an additional time burden on the radiologist; therefore, this method is not routinely used in clinical practice and is limited to only in research settings^5,14^. A recent expert-consensus panel concluded insufficient evidence to recommend any optimal method for measuring TV with MRI^1^; however volumetric measurements allows accurate assessment of tumour by adding a third dimension and can measure the maximum-diameter in any plane^15^.

PI-RADS v2.1 assessment has different decision rules for each score, and MRI derived TMD and TV were associated with csPCa^10,16^. For these reasons, tumour-size and volume estimation with MRI should be considered with care in clinical practice since it may have implications for risk stratification^12^. PI-RADS v2.1 assessment uses a 2D approach for tumour measurement, and this assessment may be influenced by 3D measurement of diameter and volume of tumour, which has not been thoroughly investigated. The objectives of the current study were to (a) develop an automated method for 3D measurement of tumour using ellipsoid-fit model, (b) compare the TV and TMD measured with the ellipsoid-fit model against the current standard i.e. the manual-assessment with axial planes by the radiologist and (c) investigate the effect of 3D measurement of tumour in PI-RADS v2.1 assessment.

## Methods

### MRI data acquisition

The current study protocol was conducted with approval from the Institutional review board (IRB), All India Institute of Medical Sciences (AIIMS), New Delhi (Reference Number: IEC-236/01.04.2016, RP-18.2016) and informed consent was waived off by IRB for this study because of the retrospective nature of the study. This study was performed in accordance with institute guidelines and regulations. MRI dataset of 43 patients (age = 65 ± 8.5 years) with clinically proven PCa (PI-RADS v2.1 score-3 = 10, score-4 = 18 and score-5 = 15) were included in this retrospective study. MRI was acquired with a 1.5 T scanner (Achieva, Philips Health Systems, the Netherlands) at the AIIMS, New Delhi with using a standard MRI protocol from August 2018 to October 2019. T2-weighted imaging (T2WI) were acquired using a turbo spin-echo sequence with TR/TE = 3330/90 ms, field of view (FOV) = 250 × 250 mm^2^, reconstructed matrix = 320 × 320, voxel size = 0.49 × 0.49 × 3 mm^2^, slice thickness = 3 mm, slice gap = 3 mm and number of slices = 36. Diffusion-weighted imaging (DWI) was acquired using echo-planar imaging sequence with TR/TE = 6831/81 ms, FOV = 292 × 292 mm^2^, reconstructed matrix = 112 × 112, voxel size = 2.6 × 2.6 × 3 mm^3^, slice thickness = 3 mm, slice gap = 3 mm, number of slice = 36 with five b-values of 0, 500, 1000, 1500 and 2000 s/mm^2^. ADC values were calculated using the vendor-provided software at the clinical workstation. A mono-exponential model using all five b-values with the least-square optimisation was used for ADC calculation^17^.

### Data processing

MRI data were transferred to a workstation (DELL Precision Tower-3620, using Intel Xeon-CPU-E3-1245-v5@3.50 GHz processor and 32 GB RAM) in DICOM format. A modified in-built routine of MATLAB (v.2018; MathWorks, Natick, MA) was used for 3D measurements^19^. The pre-processing steps included manual segmentation of the prostate gland and peripheral zone (PZ) using DWI (b = 2000 s/mm^2^), performed with the help of a radiologist (> 20 years of experience in prostate MRI). The segmented prostate region consisted of approximately 5–8 slices for each subject. Data was interpolated to generate finer slices (1 mm) and to account for slice gap. Lesion scoring was performed according to PI-RADS v2.1 guideline by a radiologist^9^.

### Measurement of diameter and volume of tumour

#### Manual-assessment by the radiologist

Initially, lesion region-of-interest (ROIs) (size range = 50–200 voxels) were drawn on PZ of DWI (b = 2000 s/mm^2^) data; then the same ROIs were later applied on T2W images and ADC images of the respective subject. In this study, maximum diameter was measured using DWI and T2W lesion ROIs on the sequence that radiologist considered to best depicted the index lesion. The ROI-based volume was calculated by the summation of all lesion areas in each slice and multiplication by the factor of slice-profile (3 mm slice-thickness plus 3 mm gap). The analysis was performed using image processing software ImageJ (v.1.48; National Institute of Health, Bethesda, USA).

#### Ellipsoid-fit model-based measurement

The ellipsoidal descriptors provide a closer approximation to irregular 3D shape of cancer lesions while preserving geometrical information as much as possible. Manually segmented 2D ROIs of the lesion previously used for radiologist assessment were stacked to form an entire 3D volume on which ellipsoid model was used to best-fit the 3D anatomy of the tumour in any direction. Because maximum diameter of the tumour might not necessarily be in the axial plane only. If *a*, *b*, and *c* are the principal semi-axes, the standard equation of an ellipsoid is

1 $$\frac{{x}^{2}}{{a}^{2}} + \frac{{y}^{2}}{{b}^{2}} + \frac{{z}^{2}}{{c}^{2}} = 1.$$

**Figure 1:**
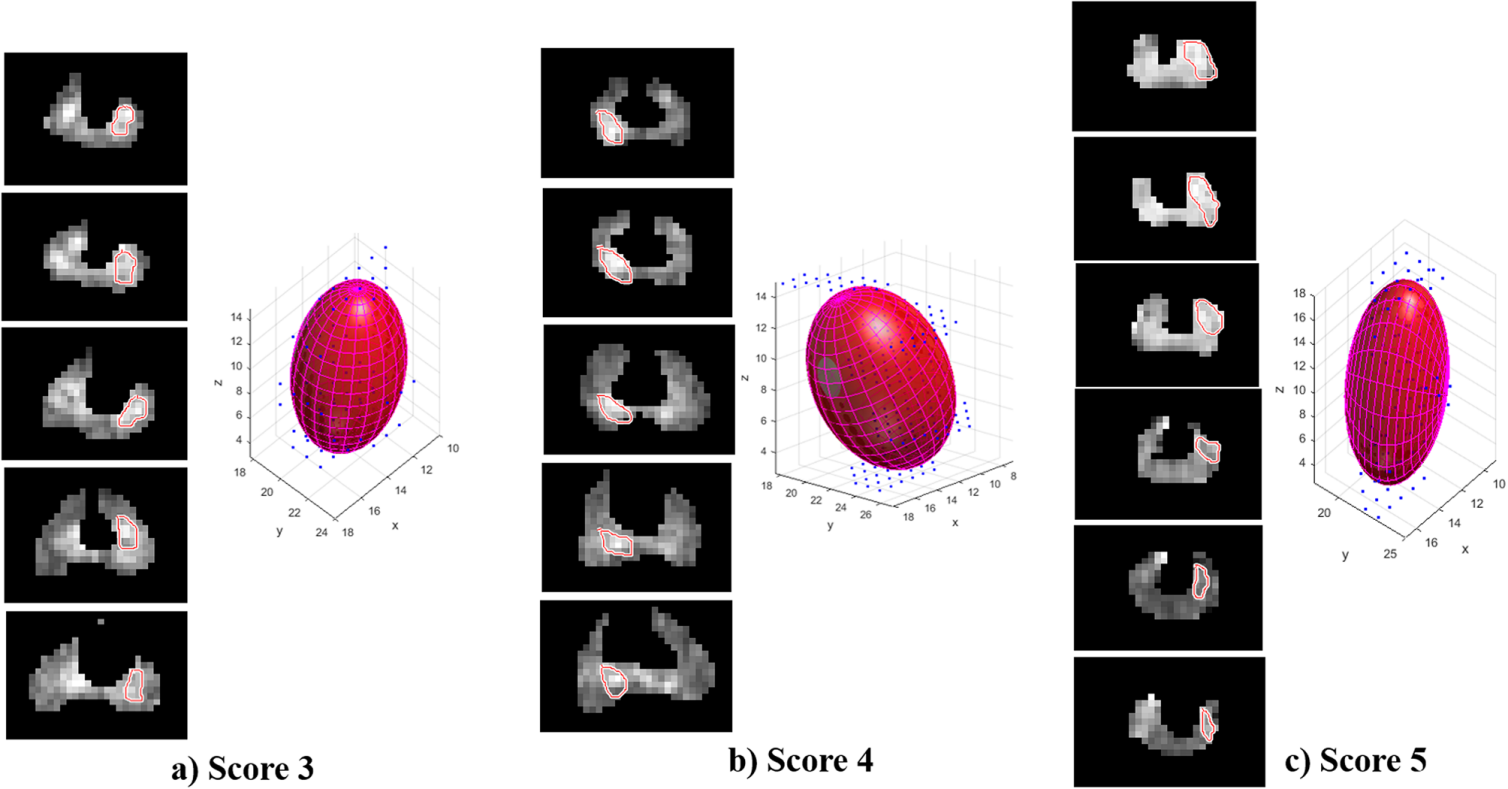
Ellipsoid fitting by 3D reconstruction from ROIs of diffusion-weighted MR images for different PI-RADS v2.1 scores, (**a**) Score 3, (**b**) Score 4 and (**c**) Score 5. The red contour in slices indicates the lesions. *ROI* Region of interest.

As shown in Li et al.^18^, a least-squares fitting of ellipsoids under the constraint *kJ* – *I*^2^ = 1 was implemented in MATLAB. where *I* = *a* + *b* + *c*, *J* = *ab* + *b*c + *ac* − *f*^2^ − *g*^2^ − *h*^2^ and *k* ~ 4 for ellipsoids with comparable semi-axes lengths. Maximum diameter was defined as the major axis of the best-fit ellipsoid and tumour volume was calculated by ellipsoid volume formula, as follows

2 $$V = \frac{4 \pi }{3}\times a\times b\times c,$$

where *a*, *b*, and *c* are the lengths of all three principal semi-axes of the ellipsoid. Figure 1 illustrates a representative example of 3D tumour reconstruction for three subjects with scores-3, 4 and 5, respectively.

### PI-RADS v2.1 assessment

MRI images were analysed and scored by the radiologist with PI-RADS v2.1 using a 5-point scale^9^. PI-RADS v2.1 score of a suspicious lesion is assessed based on signal intensity (hyperintense on high b-value DWI and hypointense on the corresponding ADC map), shape and size^9^. In the current study, PI-RADS v2.1 score ≥ 3 was defined as a visible tumour on MRI. PI-RADS v2.1 scores were assessed independently based on manual-assessment by the radiologist and 3D tumour measurements using automated ellipsoid-fit method.

### Statistical analysis

TMD and TV obtained from manual assessment were compared with ellipsoid-fit method findings using paired sample *t*-test, Pearson-correlation coefficient (*r*), Bland–Altman plots and violin-plots. The Kolmogorov–Smirnov test was used to test the normality of the data. All statistical analyses were performed using MedCalc statistical software for windows version-15.8.

## Results

**Figure 2:**
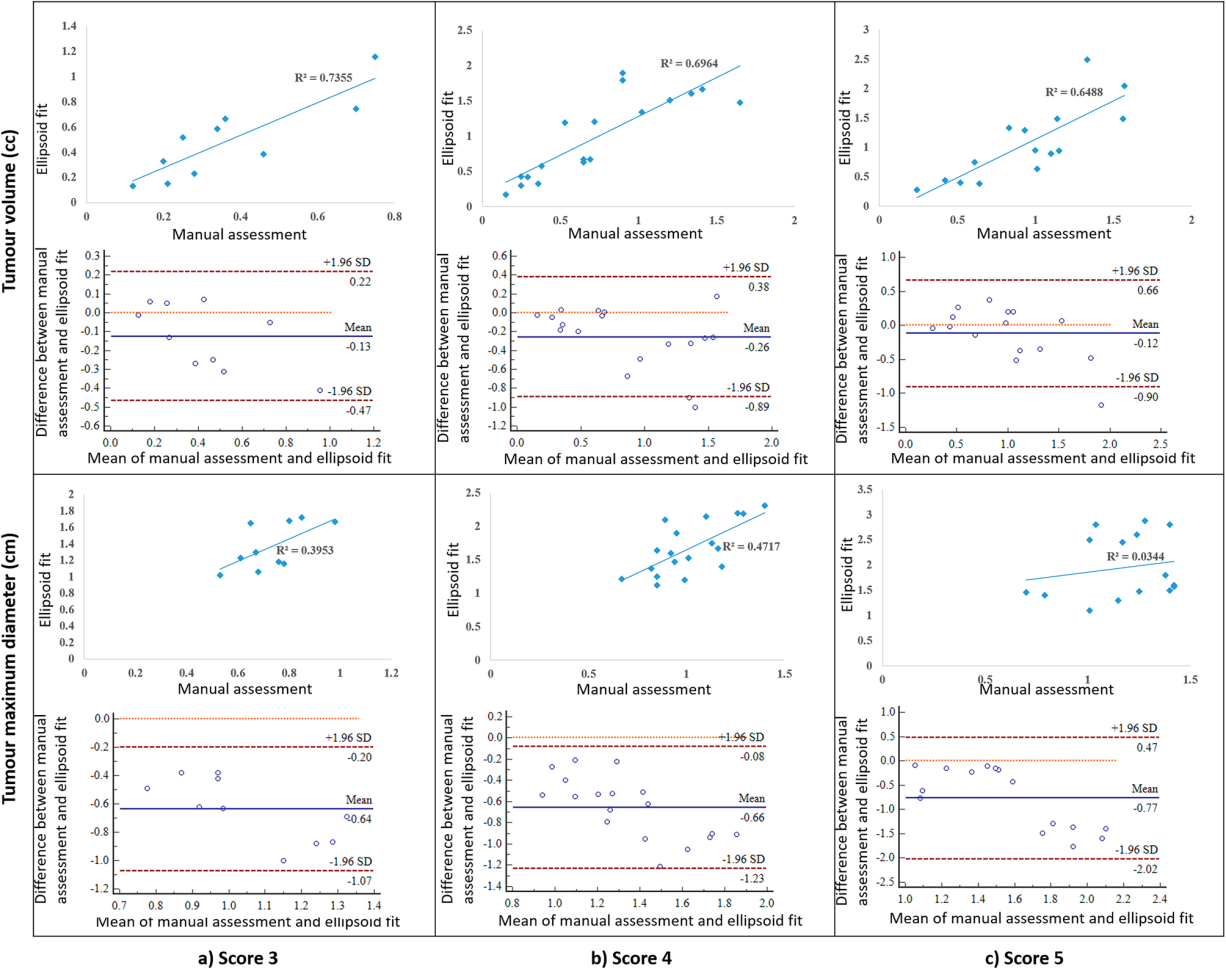
Scatter-plots and Bland–Altman plots for manual assessment and ellipsoid-fit method estimated TV and TMD for (**a**) score 3, (**b**) score 4 and (**c**) score 5. Mean difference is represented by blue solid line and limit of agreement is represented by dotted line. *TV* Tumour volume, *TMD* Tumour maximum diameter.

**Figure 3:**
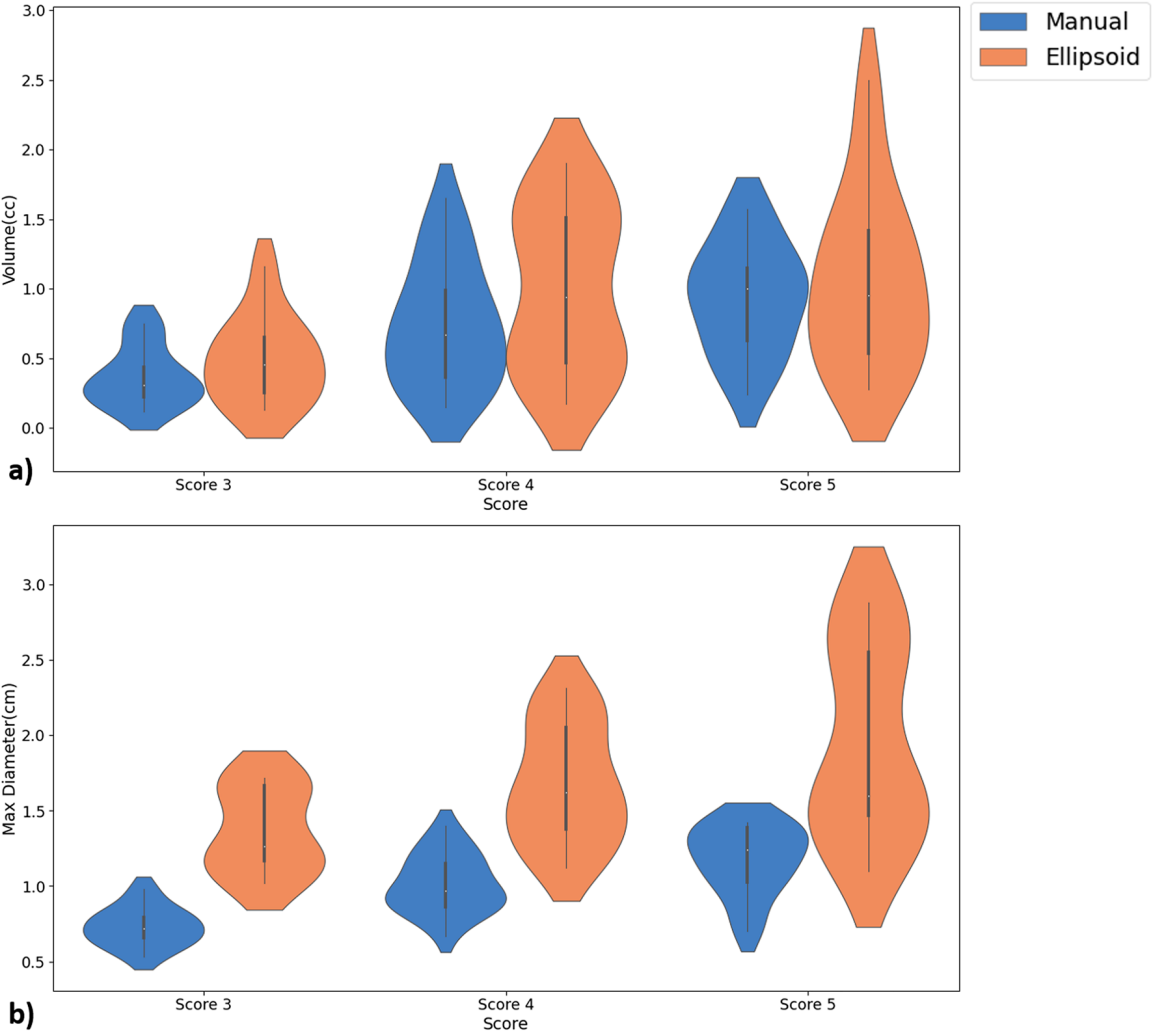
Violin plots for manual assessment and ellipsoid-fit method-based measurements of (**a**) Tumour volume and (**b**) Tumour maximum diameter for scores 3, 4 and 5.

Table 1 shows the mean ± standard deviation (SD) of TV and TMD measured with manual-assessment and automated ellipsoid-fit method for all patients with different PI-RADS v2.1 scores. The mean ± SD of TV and TMD measured by manual assessment were 0.36 ± 0.21 cc and 0.73 ± 0.12 cm for score-3, 0.74 ± 0.44 cc and 1.01 ± 0.19 cm for score-4 and 0.93 ± 0.39 cc and 1.14 ± 0.25 cm for score-5, respectively. The measurements using ellipsoid-fit method were 0.49 ± 0.31 cc and 1.36 ± 0.28 cm for score-3, 0.99 ± 0.58 cc and 1.67 ± 0.38 cm for score-4 and 1.12 ± 0.78 cc and 1.97 ± 0.67 cm for score-5, respectively. Ellipsoid-fit based 3D-measurement showed significantly higher values (*p* < 0.05) for both TV and TMD than manual assessment by factor of approximately 1.31 for TV and 1.74 for TMD. There was good correlation (*r* = 0.85 and *p* < 0.05) for TV and moderate correlation (*r* = 0.55 and *p* < 0.05) for TMD between manual and ellipsoid-fit measurement method. A significant bias in the agreement between manual assessment and ellipsoid-fit based tumour measurements, graphically displayed by scatter and the Bland–Altman plots (Fig. 2). It was observed that manual assessment of TV and TMD were lower by a range of 17–46% for all scores compared to ellipsoid-fit based measurements. Figure 3 shows the violin-plots, representing the distribution of TV and TMD across all patients with different PI-RADS v2.1 scores for manual assessment and ellipsoid-fit method.

**Table 1:** Tumour volume and maximum-diameter for different PI-RADS v2.1 scores, in terms of mean ± standard deviation (SD); SD was calculated across all subjects within each score.

Scores	Volume (cc)		Maximum diameter (cm)	
	Manual assessment	Automated ellipsoid fit	Manual assessment	Automated ellipsoid fit
Score 3	0.36 ± 0.21	0.49 ± 0.31	0.73 ± 0.12	1.36 ± 0.28
Score 4	0.74 ± 0.44	0.99 ± 0.58	1.01 ± 0.19	1.67 ± 0.38
Score 5	0.93 ± 0.39	1.05 ± 0.78	1.14 ± 0.25	1.97 ± 0.67

The absolute relative percentage difference between manual-assessment and ellipsoid-fit measurement derived TV and TMD was 26% for score-3, 25% for score-4, 17% for score-5 and 46% for score-3, 34% for score-4 and 42% for score-5, respectively. 3D tumour measurements based PI-RADS v2.1 assessment showed that for score 3, 4 out of 10 patients (40%) were classified as score-5, and for score-4, 11 out of 18 (61%) patients were classified as score-5 compared to manual-assessment.

**Figure 4:**
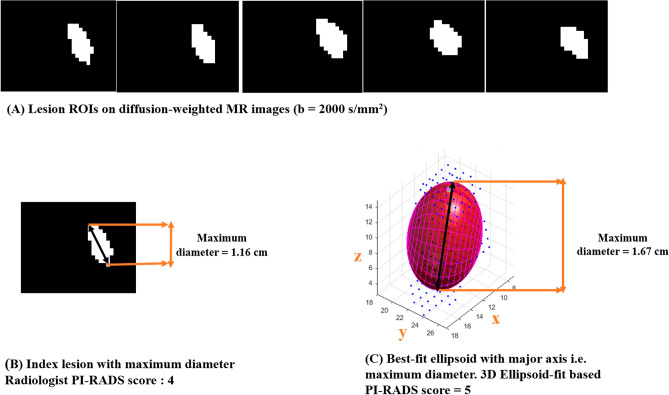
Lesion measurements of a representative patient (age = 60 years) using manual and ellipsoid-fit method. (**A**) Lesion ROIs, (**B**) Radiologist manual lesion measurement and (**C**) 3D ellipsoid-fit method based lesion measurement.

Figure 4 represents the lesion measurement of one representative male patient (age = 60 years) using manual and ellipsoid-fit method, where PI-RADS score changed from 4 to 5. Table 2 presents the TV and TMD for all 15 patients where PI-RADS score was found to be changed among the two methods; it clearly shows the increase in PI-RADS score from 3 to 5 in 4 patients and from 4 to 5 in 11 patients in the cohort (Table 2).

**Table 2:** PI-RADS score assessment using manual and automated 3D ellipsoid fit approach for (A) Score 3 and (B) Score 4 patients which classified as score 5. *TMD* Tumour maximum diameter, *TV* Tumour volume.

Subjects	Manual assessment			Automated 3D ellipsoid fit		
	TV (cc)	TMD (cm)	Score	TV (cc)	TMD (cm)	Score
**(A)**						
1	0.46	0.98	3	0.39	1.67	5
2	0.75	0.85	3	1.16	1.72	5
3	0.70	0.80	3	0.75	1.68	5
4	0.36	0.65	3	0.67	1.65	5
**(B)**						
1	0.72	0.92	4	1.21	1.60	5
2	1.02	1.16	4	1.35	1.67	5
3	0.53	1.29	4	1.20	2.19	5
4	1.34	1.40	4	1.61	2.31	5
5	0.65	1.01	4	0.63	1.53	5
6	0.90	1.26	4	1.90	2.20	5
7	0.65	1.13	4	0.68	1.75	5
8	0.29	0.85	4	0.42	1.64	5
9	1.20	0.95	4	1.52	1.90	5
10	1.65	1.10	4	1.48	2.15	5
11	0.90	0.89	4	1.80	2.10	5

## Discussion

Accurate non-invasive measurement of prostate TV could significantly improve the determination of tumour prognosis. MRI has become a standard method for patient selection and guiding biopsy and focal therapy^5^. Maximum-diameter and volume of tumour provide an important information in determining the clinically significant PCa^2,3^.

Several studies have evaluated the agreement between MRI based TV and histological TV based on radical prostatectomy specimens and found an underestimation of MRI based measurement, ranging from 4 to 97%^5,11^. Wolters et al. reported a positive relationship between TV of > 0.5 cc at radical prostatectomy, PCa staging and Gleason-score^19^. Another study found that in 2D measurement, TMD was a valuable parameter to differentiate between PI-RADS assessment score-4 and 5, and showed a significant correlation with higher Gleason scores^2^. At present, there are no other feasible non-invasive image-based alternatives, although developments in new methods can refine volume estimations. PI-RADS v2.1 aims to standardise the estimation of prostate gland volume by proposing either whole prostate segmentation or the use of the ellipsoid-fit method^9^. In some studies, ellipsoid-fit method was used to calculate prostate gland volume to assess the diagnostic accuracy of prostate-specific antigen density (PSAd) in predicting PCa, which provided greater reliability than the alternative methods^20,21^; it may be preferable to use the ellipsoid-fit method to determine the 3D measurement of prostate tumour to assess the PI-RADS v2.1 scores, as proposed in the current study.

In the current study, an automated ellipsoid-fit model was used for 3D measurement of TV and TMD and these quantitative parameters were compared with the manual assessment of the radiologist using the current standard axial slices. Along with this, the effect of 3D measurement of tumour in PI-RADS v2.1 assessment was also investigated. Maximum diameter and volume of the tumour in PZ were measured from DWI and T2WI, using the sequence that radiologist considered the best depicted the index lesion. Only biparametric MRI (T2WI and DWI) was used for this study but inclusion of additional sequences might improve the diagnosis, which need to be explored. There might be some variations in TV and size depending on the type of imaging technique as lesion contrast may vary in different sequence^22^.

The current study found that ellipsoid-fit method showed higher TV and TMD values than manual-assessment with variability ranging from 17 to 26% and 34 to 46%, respectively. It was observed that ellipsoid-fit based TMD showed large variation than manual-assessment, which was expected as radiologist measurement are carried on axial slices showing largest lesion. However, during ellipsoid-fit, major axis is considered as TMD, which is expected to be higher than 2D measurement. Score-3 measurements were found to have higher variability than score-4 and score-5, due to intermediate density and heterogeneous tumour morphology which remains obscure on prostate MRI.

Bland–Altman analysis showed a significant bias in the agreement between manual-assessment and ellipsoid-fit based measurements. The bias might occur due to the conservative approach of radiologist for tumour measurements and the inherent curvature bias in 3D ellipsoid-fitting^23^. In literature, studies have found an underestimation of TV (4 to 97%) by a radiologist based on 2D assessment with MRI against histopathological measurements, similarly in this study where manual-assessment by radiologist was found to underestimate TV by 17–26% and TMD by 34–46% compared to the ellipsoid-fit based measurements. From the results of the current study, it is implied that the measurements obtained with ellipsoid-fit method for PCa can be comparable with the literature histopathological analysis. Violin-plot was utilised in this study to visualise the distribution of TV and TMD across all patients as well as its probability density.

As found in the current study with the ellipsoid-fit method based measurements, 61% patients of score-4 and 40% patients of score-3 were changed as score-5 because of higher TMD (≥ 1.5 cm) as per updated PI-RADS v2.1. PI-RADS v2.1 scores were found to have a linear relationship with TV, which could be an important clinical parameter for PCa diagnosis. The current study only demonstrates that maximum diameter of tumour can be practically in any direction, not necessarily in axial plane only. Thus obvious hypothesis was whether measuring the maximum diameter in these specific cases, changes the PI-RADS score. The current study focuses on the development of 3D measurement tool using ellipsoid-fit approach and give only preliminary evidence for its impact on PI-RADS v2.1 score in small patient cohort. By observing the results, it is evident that ellipsoid-fit based measurements were higher than the conventional radiologist assessment in some cases, which has changed the PI-RADS scores. One of the major limitations of this study is the Gleason scores based on radical prostatectomy specimen were not available at our centre and biopsy proven Gleason scores were available but not for all the patients for building a conclusive evidence. There was also much variability in the Gleason scores that were found in patients under this study, even in patients whose PI-RADS scores were not changed by ellipsoid method. This is just an observational evidence. The bigger question to embark upon is for the PI-RADS community to evaluate this 3D tumour measurement with the larger studies to evaluate the clinical implications, which are beyond the scope of this study.

There are few artificial intelligence (AI)-based automatic segmentation tool which can delineate lesions and calculate the lesion volume. A comprehensive overview of current commercial products/public tools is summarized by Sunoqrot et al.^24^ and these tools have shown good diagnostic accuracy for PCa among all available AI-based tool^24,25^. However, there are some inherited problems these methods usually face, such as it is not always possible to get access to these trained models for larger clinical adoption, and often it is not simple for the end users to implement these. The larger question on bias in training dataset with respect to MRI machine, acquisition site and populations cohort etc., always requires a wider multi-centric and multi-geographical location dataset to be included in the training process, which is currently not available.

Our study focuses on demonstrating the development of a 3D measurement tool using a simple mathematical operation, an ellipsoid-fit approach and its impact on PI-RADS v2.1 score. The program is developed in-house for lesion measurement in the MATLAB (Math Works Inc., v2018, Natick, MA), which gave us insightful information of whole fitting algorithm and PI-RADS assessment. It is easily scalable and require less computation power as compared to any AI-based approach.

There are other limitations to the current study. First, the data were acquired from a single institution and a small cohort; it may influence the outcomes. A large cohort and multicentre study can provide stronger evidence for larger clinical application. Second, the reference measurement was done by only one radiologist; inter-observer and intra-observer variability were not evaluated. Third, benign lesions (score-2) were not included in this study because these cases are not usually biopsied as per current standard of guidelines. It is also observed that, not all lesions fit well with the ellipsoid model, especially the PI-RADS 5. This could be possibly because the extra prostatic invasion of the lesion was not evaluated in this study and the inherent curvature bias in ellipsoid-fitting methodology^23^, which need to be further investigated.

## Conclusion

3D measurement of tumour using automated ellipsoid-fit method has been developed and its impact on PI-RADS v2.1 scoring was assessed in a cohort of patients with prostate cancer. Ellipsoid-fit method led to find an increase in the TMD by 34–46% and TV by 17–26% and more than 40% of patients with PI-RADS v2.1 score-3 or 4 were changed to score-5. In future, a study on evaluating the potential clinical implications of proposed 3D ellipsoid fit based tumour measurements in PI-RADS scoring should be carried out.

## Data Availability

The datasets generated during and/or analysed during the current study are not publicly available due to privacy or ethical restrictions but are available from the corresponding author on reasonable request.
